# Growth Performance, Antioxidant Activity, Immune Status, Meat Quality, Liver Fat Content, and Liver Histomorphology of Broiler Chickens Fed Rice Bran Oil

**DOI:** 10.3390/ani11123410

**Published:** 2021-11-29

**Authors:** Shaimaa Selim, Eman Hussein, Nazema S. Abdel-Megeid, Sahar J. Melebary, Mohammad S. AL-Harbi, Ahmed A. Saleh

**Affiliations:** 1 Department of Nutrition and Clinical Nutrition, Faculty of Veterinary Medicine, University of Menoufia, Shibin El-Kom 32514, Egypt; 2 Department of Poultry and Fish Production, Faculty of Agriculture, University of Menoufia, Shibin El-Kom 32514, Egypt; eman.hussien@agr.menofia.edu.eg; 3 Department of Cytology and Histology, Faculty of Veterinary Medicine, University of Sadat City, Sadat City 32897, Egypt; nazemah.abdelmageed@vet.usc.edu.eg; 4 Department of Biology, College of Science, University of Jeddah, Jeddah 21493, Saudi Arabia; Sjmelebary@uj.edu.sa; 5 Department of Biology, College of Science, Taif University, P.O. Box 11099, Taif 21944, Saudi Arabia; mharbi@tu.edu.sa; 6 Department of Poultry Production, Faculty of Agriculture, Kafrelsheikh University, Kafrelsheikh 33516, Egypt; ahmed.saleh1@agr.kfs.edu.eg

**Keywords:** performance, carcass traits, blood biochemical constituents, abdominal fat color, meat fatty acid profile, liver histology, broilers

## Abstract

**Simple Summary:**

There are numerous approaches for enrichment of broiler’s meat with valuable nutrients, for instance the enrichment with polyunsaturated fatty acids (PUFA). The addition of vegetable oils in the diets of broilers is an appropriate strategy to enrich the chicken meat with beneficial FA, however, this enrichment is accompanied by a lipid peroxidation with a resultant decrease in the nutritional value, quality, and shelf-life of the meat, and for that reason, the dietary supplementation with antioxidants becomes necessary. What places rice bran oil (RBO) on top of other vegetable oils is its antioxidant components and unique fatty acid profile and it is reported to induce substantial lipid-reducing effects and antioxidant properties. Therefore, this study was performed to determine the influence of RBO inclusion in the diets of broiler chickens on performance, carcass characteristics, blood parameters, meat quality, antioxidant activity, liver lipid content, and liver histological structure. RBO inclusion had a positive effect on the growing performance, dressing percentage, and immune status. Furthermore, RBO supplementation decreased the abdominal fat yield and EE content in the meat, while it increased the content of PUFA in the meat, which may be beneficial for consumers. RBO improved the antioxidant capacity of the meat and the liver, whereas it reduced the concentration of cholesterol and triglycerides in the blood, meat and liver. RBO could be used as an efficient ingredient in broiler chickens’ diets to improve performance, immune status, antioxidant activity, blood lipid profile, and the nutritive value of meat.

**Abstract:**

This trial was performed to determine the effect of rice bran oil (RBO) inclusion in diets of broiler chickens on performance, carcass characteristics, blood parameters, meat quality, antioxidant activity, liver lipid content, and liver histological structure. The 35-day feeding trial was conducted on 240 one-day-old Ross 308 broiler chickens, allocated to four treatment groups with six replicates each. RBO was examined at different inclusion levels, 0% (control), 1% (RBO_1%_), 1.5% (RBO_1.5%_), and 2% (RBO_2%_) in a completely randomized design. The results showed that at the end of the trial (35 days) the RBO supplementation had positive effects (*p* < 0.001) on the productivity parameters, but the feed intake was linearly decreased due to RBO inclusion. In addition, RBO supplementation linearly improved (*p* < 0.05) the dressing percentage, breast yield, immune organs relative weights, and meat glutathione concentration, while it decreased (*p* < 0.01) the abdominal fat yield and meat crude fat, triglycerides, cholesterol, and Malondialdehyde (MDA) contents in broiler’s meat. Moreover, serum total protein, globulin, and high-density lipoprotein contents improved noticeably (*p* < 0.01) due to offering an RBO-supplemented diet, but serum total lipids, total cholesterol, triglyceride, low-density lipoprotein, and aspartate aminotransferase concentrations linearly reduced (*p* < 0.01). The RBO supplementation augmented (*p* < 0.05) the phagocytic index, phagocytic activity, and antibody titer compared to control. On the other hand, RBO inclusion had no effect on the breast, thigh, or abdominal fat color parameters. Moreover, RBO supplementation reduced (*p* < 0.01) the content of total saturated FA (SFA), but increased (*p* < 0.01) the content of total monounsaturated FA (MUFA), and polyunsaturated FA in both breast and thigh meat. Chemical analysis of the liver tissue samples revealed that the inclusion of RBO linearly reduced (*p* < 0.05) hepatic cholesterol, triglyceride, and MDA contents. Histologically, the lipid percentage and number of lipid droplets (*p* < 0.01) were markedly lessened in the RBO-supplemented groups. The histological structure of the liver asses by light and electron microscope were normal in all groups without any pathological lesions. It is concluded that RBO could be used as a valuable ingredient in broiler chickens’ diets to stimulate the growing performance and immune status, enhance the antioxidant activity and blood lipid profile, augment liver function, and improve the nutritive value of the meat.

## 1. Introduction

A remarkable increase in worldwide meat consumption has been recorded during the last few decades, with a noteworthy increase in developing countries. The global production of chicken meat has been raised from 100.46 million tonnes (Mt) in 2014 to 118.02 Mt in 2019 [1]. Compared to other meat types, chicken meat is steadily and continuously raising worldwide owing to its low-price, health benefits, and sustainable production [2]. Meat quality is considered a valuable assessment criteria associated with the prerequisites that must be met to accomplish the consumers’ demands and expectations. Consumers of the 21st Century are highly demanding ones with greater concern about meat quality, safety, and health benefits [3], which in turn necessitates the development of new approaches to enhance the meat nutritive value [4]. Currently, nutritional approaches are mainly depending on the application of natural constituents in poultry diets to enhance the nutritive value of the poultry products for better human health [2,3,4].

There are numerous approaches for enrichment of broiler’s meat with valuable nutrients, for instance the enrichment with polyunsaturated fatty acids (PUFA). PUFA (ω3 and ω6 fatty acids, FA) are beneficial for the proper functions of the body, but these fatty acids cannot synthesize in the body and must be supplied by the diets. The addition of vegetable oils in the diets of broilers is an appropriate strategy to enrich the chicken meat with beneficial FA [5], which is in concordance with the consumers’ interest and immunity perspectives [5,6,7,8]. However, enrichment of broiler’s meat with PUFA is accompanied by a great susceptibility to lipid peroxidation with a resultant decrease in the nutritional value, quality, and shelf-life of the meat [5,8], and for that reason, the dietary supplementation with antioxidant becomes necessary [5]. Synthetic antioxidants are included in the diets of poultry to avoid or limit lipid peroxidation in the meat products [9]. Due to the consumers’ awareness about consuming natural feed ingredients, nutritionists are seeking for natural antioxidants to replace synthetic ones [5,10]. These natural antioxidants have numerous advantages for instance lowering the incidence of metabolic diseases and improving the shelf life of meat, thus optimizing food safety and security [5].

Rice bran oil (RBO) is receiving great interest among other conventionally vegetable oils owing to its good quality, extended shelf-life, and well-proportioned fatty acid composition as well as the presence of numerous antioxidant components [11,12]. RBO is rich in tocopherols, tocotrienols, and other bioactive phytochemicals, including phytosterols, γ-oryzanol, squalene, and triterpene alcohols [13,14]. These natural bioactive components have been reported to reveal antioxidant, anti-inflammatory, and hypocholesterolaemic properties as well as boost the immune response [12]. RBO is one of the healthful edible oils because of its balanced fatty acids profile, with a ratio of 0.6:1.1:1 for saturated fatty acid (SFA), monounsaturated fatty acid (MUFA), and PUFA [11]. Oleic acid (C18:1; 42%), linoleic acid (C18:2; 32%), and palmitic acid (C16:0; 20%) are the three main fatty acids in RBO and represent about 90% of the total fatty acids in RBO [11,12]. Even though RBO has small proportion of α-linolenic acid, it is sufficient for de novo synthesis of other ω3-PUFA such as eicosapentaenoic acid and docosahexaenoic acid in tissue phospholipids [15]. 

What places RBO on top of other vegetable oils is its antioxidant components, which have been documented to have an outstanding nutritive significance and are recognized to induce substantial lipid-reducing effects and antioxidant properties based on research performed on mice and humans (see review, 12). Previous studies on the dietary supplementation of RBO in broilers showed enhanced growth performance, improved immune response, and reduced cholesterol concentration [16,17]. However, data concerning the impacts of dietary inclusion of RBO in the diets of broiler chickens on the antioxidant capacity, meat quality, meat fatty acid composition, hepatic lipid profile, and liver histomorphology are limited. Therefore, the present study aimed to investigate the effects of graded dietary inclusion levels of RBO on the growth performance, carcass traits, blood biochemical parameters, meat quality, abdominal fat color, lipid peroxidation, liver lipid content, and liver histological structure of broiler chickens. We hypothesized that the dietary inclusion of RBO might improve the meat quality, enhance the antioxidant capacity, and decrease meat and liver lipid content of broiler chickens owing to its unique FA profile and antioxidant constituents.

## 2. Materials and Methods

### 2.1. Ethical Approval

The care and procedure used for broiler chickens of the current trial were permitted by Institutional Animal Care and Use Committee (IACUC), Faculty of Veterinary Medicine, University of Sadat City (Ethical approval number: VUSC-018-1-20). The trial was complied with the European Union Council Directive 98/58/EC and Directive 2010/63/EU on the protection of animals used for experimental and other scientific purposes.

### 2.2. Experimental Design and Diets

This trial was performed on 240 one-day-old Ross 308 mixed sex broiler chickens (equal distribution between males and females in each group), obtained from a local commercial hatchery (Alwadi co., Sadat City, Menoufia, Egypt). Different levels of rice bran oil (RBO) were studied at 0% (control), 1% (RBO_1%_), 1.5% (RBO_1.5%_), and 2% (RBO_2%_) by replacing the vegetable oil (soybean oil) from the control basal diet. The broiler chickens were individually weighed and randomly allotted to four treatment groups of 60 broiler chicks each (six replicates/group) using a completely randomized design. The experiment lasted for 35 days and was allotted to three periods: starter (from 1–10 days), grower (from 11 to 24 days), and finisher periods (from 25 to 35 days). The experimental diets were formulated to be isocacloric and isonitrogenous and in accordance with Ross 308 broiler nutrition specifications [18]. Ingredients and chemical composition of the diets are presented in Table 1. Rice bran oil tested in the current experiment was obtained from Agricultural Research Center, Egypt. The fatty acid composition and γ-oryzanol of RBO and soybean oil are presented in Table 2. Feed and water were offered *ad libitum*. All broiler chicks were kept under the same management, hygienic, and environmental conditions. Broiler chickens in each replicate were housed in floor pens on wood shaves litter, at an optimum stocking density of 30 kg/m^2^. The temperature and lighting programs were consistent with the recommendations of Ross breeding guide. The light schedule in all trial pens was kept at 23 L:1 D h for the first 7 days, followed by 20 L:4 D h till the end of the trial. The initial ambient temperature was approximately 32 °C during the first 7 days of life and then gradually decreased 2 °C per week till it reached 22 °C. The relative humidity was kept between 65 and 75%. No mortality was noted during the overall experimental period. The vaccination program was performed under the supervision of a veterinarian.

### 2.3. Broiler Performance and Carcass Traits 

Throughout the trial period (1–35 days), the subsequent parameters were recorded: live weight (LW, g), weight gain (WG, g/chick), feed intake (FI, g feed/chick) and feed conversion ratio (FCR, g feed/g weight gain). Live weights of broiler chickens were recorded weekly by weighing the broiler chickens individually and FI was recorded on a daily basis. Average WG, FI, and FCR were calculated for the starter, grower, and finisher periods. At the end of the feeding trial period (35 days), 12 broiler chickens (six males and six females) of an average LW of each group were selected for slaughtering by cervical dislocation after a period of 12h fasting. The dressed carcass, breast and thigh yields, liver, gizzard, heart, spleen, thymus gland, bursa of Fabricius, and abdominal fat as a percentage of LW were recorded. 

### 2.4. Sample Collection

At the end of the experiment (35 days), broilers were kept for blood sampling from the jugular vein, and then euthanized by cervical dislocation. After that, the breast and thigh meat samples were collected, divided into several parts, kept in plastic bags, labelled, and allocated for the subsequent analyses, involving pH determination, color measurements, proximate chemical analysis, lipid profile, lipid peroxidation, and fatty acid composition. Liver samples were also distributed into several parts for further determinations, including cholesterol and triglycerides concentrations, MDA, and histomorphology.

### 2.5. Blood Biochemical Parameters

At day 35, the collected blood samples were allowed to coagulate by centrifugation at 3000 rpm for 15 min. The serum samples were collected and kept at −20 °C for the clinical and biochemical analyses, involving protein and lipid metabolites, liver and kidney functions, and antibody titers. The serum biochemical constituents were analyzed spectrophotometrically (ultraviolet spectrophotometer UV4802, Unico Co., Dayton, OH, USA) using commercial analytical kits (Spectrum Diagnostics, Al Obour, Cairo, Egypt) following the manufacturer’s manuals. Hemagglutination inhibition (HI) test was used to determine the antibody responses as described by Takatasy [19]. Another set of blood samples (12 blood samples/group) was collected into sterile vials having anticoagulant to determine phagocytic activity and phagocytic index, in accordance with the method of Kawahara et al. [20].

### 2.6. Meat Quality Measurements

The pH of the meat samples (breast and thigh) was measured in triplicate using a pH-meter (Beckman model 350, Beckman Coulter, Inc., East Lyme, CT, USA) at 24 h post-mortem as described by Egan et al. [21]. Measurements of meat samples and abdominal fat color parameters were performed at 24 h post-mortem by recording the following parameters: L* (lightness), a* (redness), and b* (yellowness) following the Commission International de l’Eclairage [22] using a model CR 410 Chroma meter (Konica Minolta, Tokyo, Japan) and documented the average of three measurements per each meat sample. Breast and thigh muscles were used in triplicate for measuring the proximate chemical analysis, including moisture (Method 950.46), crude protein (CP; Method 981.10), ether extract (EE; Method 960.39), and ash % (Method 920.153) following the procedures of AOAC [23].

Samples of breast and thigh meat were preserved in the refrigerator at a temperature of 4 °C for 7 days before measuring the lipid peroxidation. Malondialdehyde (MDA) was measured spectrophotometrically (UV4802, Unico Co., Dayton, OH, USA) following the method described by Ohkawa et al. [24] by using the analytical kits (Spectrum Diagnostics, Al Obour, Cairo, Egypt), and expressed as nmol/g of the meat. Glutathione peroxidase (GPx) activity was measured in accordance with Paglia and Valentine [25] using commercial analytical kits (Spectrum Diagnostics, Al Obour, Cairo, Egypt), and expressed as U/g of the meat. The meat cholesterol and triglyceride concentrations were performed spectrophotometrically (UV4802, Unico Co., Dayton, OH, USA) in accordance with the method of Bohac and Rhee [26]. The meat lipids were extracted according to Folch et al. [27] for FA analyses. The assessments of FA in the breast and thigh meat were done via the transformation of EE to FA methyl esters [28] using a gas chromatograph (Model GC-14A, Shimadzu Corporation, Kyoto, Japan) with a flame-ionization detector and a polar capillary column (BPX70, 0.25; SGE Incorporated, CA, USA).

### 2.7. Liver Lipid Content, MDA, and Histomorphology

The hepatic total cholesterol and triglycerides levels were measured using the analytical kits (Sigma-Aldrich St. Louis, MO, USA) and performed as per the manufacturer’s manuals. Liver MDA levels were spectrophotometrically (UV4802, Unico Co., Dayton, OH, USA) determined according to Ohkawa et al. [24] using the analytical kits (Spectrum Diagnostics, Al Obour, Cairo, Egypt), and expressed as mg/kg of the liver.

For light microscopy, a liver sample (1 cm^2^) was excised from each broiler chicken, immediately fixed in 10% neutral buffered formalin for 48 h. Afterwards, the samples were dehydrated in ascending grades series of alcohol, cleared in methyl benzoate, and embedded in paraffin wax. Sections of 5–7 µm thickness were performed using rotatory microtome and stained with Harri′s Hematoxylin and eosin (H & E) for the routine histological examination [29]. The photomicrographs were taken using Leica digital camera connected with bilocular microscope for better demonstration of the results. For quantitative histological analyses, the hepatic lipid content was evaluated by assessing the percentage of the area occupied by lipid droplets inside the liver parenchyma with circularity filter to exclude artifacts such as sinusoids. 

For electron microscopic examination, the liver samples were fixed in 2.5% buffered glutaraldehyde in 0.1 M phosphate buffer solution (pH 7.4) at 4 °C for 2 h, washing three times with phosphate buffer solution (PBS) (10 min, each), post fixed in 1% Osmic acid for 30 min, washing three times with PBS (10 min each), then dehydrated with ascending series of ethyl alcohol (30, 50, 70, 90% and absolute alcohol) infiltrated with acetone, for 30 min. After dehydration, samples were embedded in Araldite 502 resin. The plastic molds were cut in the Leica ultra-microtome, stained with 1% toluidine blue and photographed. After examination of semi-thin sections, ultra-thin sections were cut, stained with uranyl acetate. Then, counter stained with lead citrate [30]. The ultrathin (80 nm) sections were examined with a transmission electron microscope (JEOL-JEM-100 SX, Tokyo, Japan). For each broiler chicken, the diameters of 40 lipid droplets, the number and area of the lipid droplets in 10 randomly selected fields were determined manually. The number of lipid vacuoles in 1 mm^2^ of the hepatic section as well as the area of the lipid droplets as a percent were measured. 

### 2.8. Statistical Analysis

The trial was performed using a completely randomized design with four experimental groups of six replicates each. The normality of the data was checked by Kolmogorov Smirnov test before the statistical analysis. Data were analyzed by One-way ANOVA using IBM SPSS statistical package (version 22, SPSS Inc., Chicago, IL, USA) to evaluate the effect of treatment, along with a Tukey’s test (*p* < 0.05). The replicate pen was used as an experimental unit for the performance data and the bird for the other variables. Orthogonal polynomial contrast test for linear, quadratic, and cubic to estimate the effects of RBO supplementation. Significance was set at *p* < 0.05 and values are presented as means ± standard error of the mean (SEM).

## 3. Results

### 3.1. Broilers Growth Performance

Growth performance data of broiler chickens fed the control and graded levels of RBO diets are shown in Table 3. Broiler chickens consuming the 1.5 and 2% RBO diets had a significantly higher (*p* < 0.001) LW during the starter and grower periods compared with those consuming the control diet, this was supported by linear and quadratic effects (*p* < 0.001) due to the inclusion of RBO in the diets of broiler chickens. Positive total weight gains (*p* < 0.001; linear, *p* < 0.001) were achieved for broiler chickens consuming the RBO diets than those fed on the control diet. However, broiler chickens fed the RBO diets had a significantly (*p* < 0.001; linear, *p* < 0.001; quadratic, *p* < 0.001) lower FI compared with those consuming the control diet. The mean FCR of the RBO treatment groups was significantly improved (*p* < 0.001) during the starter, grower, and whole the experimental periods compared to the control group, this was indicated by linear (*p* < 0.001) and quadratic (*p* < 0.001) responses as a result of the inclusion of RBO in their diets.

### 3.2. Carcass Traits

Carcass traits of broiler chickens are shown in Table 4. The inclusion of RBO in the diets of broiler chickens influenced the dressing per cent, with significantly greater values (*p* < 0.001; linear, *p* < 0.001; quadratic, *p* < 0.05; cubic, *p* < 0.01) being observed in the RBO group compared to the control group. RBO inclusion to the broiler diets at levels of 1.5 and 2% led to an increase (*p* < 0.001; linear, *p* < 0.05; quadratic, *p* < 0.01; cubic, *p* < 0.001) in the breast meat yield compared to those fed the control and RBO_1%_ diets. Non-significant difference (*p* > 0.05) was noted for the thigh, liver, gizzard, or heart yields between the treatment groups. However, the relative weight of abdominal fat decreased (*p* < 0.05; linear, *p* < 0.01, quadratic, *p* < 0.01) with the dietary inclusion of RBO compared to control. The immune organs’ relative weights were significantly increased (*p* < 0.01) in the RBO groups compared to the control group, this was supported by linear response (*p* < 0.001).

### 3.3. Blood Parameters

The serum biochemical parameters of the broiler chickens fed the control and graded levels of RBO are presented in Table 5. Serum total protein and globulin showed linear (*p* < 0.00) and quadratic (*p* < 0.05) responses to the increasing dietary level of RBO, with a maximum corresponding to the RBO_1.5%_ and RBO_2%_ groups (*p* < 0.01), while there was non-significant difference in serum albumin concentration among the treatment groups. The serum concentrations of total lipids and triglycerides were linearly and quadratically (*p* < 0.001) decreased in the RBO groups compared to the control group. The RBO-supplemented groups exhibited significantly lower (*p* < 0.001) serum total cholesterol compared to control; this was characterized by linear (*p* < 0.001) and quadratic (*p* < 0.05) responses due to the inclusion of RBO in the diets of broiler chickens. RBO supplementation to broiler diets led to an increase (*p* < 0.001) in the serum HDL concentration and a decrease (*p* < 0.05) in the serum LDL level compared to the control group.

Serum AST showed linear and quadratic responses (*p* < 0.05) to the increasing dietary RBO levels, with lower values being noted for the RBO_1.5%_ and RBO_2%_ groups, while only a linear response (*p* < 0.05) for lower serum ALT and ALP concentrations were recorded. Serum creatinine and uric acid concentrations did not significantly vary (*p* > 0.05) among dietary treatments. The dietary inclusion of RBO did not affect the HI test for infectious bronchitis virus (HIBB) or bursa disease (HIBD). However, the RBO-supplemented groups exhibited significantly greater (*p* < 0.001) antibody titers to Newcastle Disease (HIND) compared to the CON group, this was indicated by a linear response (*p* < 0.001). Furthermore, a significant increase (*p* < 0.05; linear, *p* < 0.05) was also observed for the phagocytic activity and phagocytic index in the groups that included RBO in their diets compared to control.

### 3.4. Meat Quality Traits

Table 6 and Table 7 showed the color parameters, pH, proximate chemical composition, and antioxidant capacity data of breast and thigh meat, respectively. Overall, there were non-significant differences in pH, L*, a*, or b* of breast and thigh meat of broiler chickens fed the RBO diets compared with those fed the control diet. The inclusion of RBO to the diets of broiler chickens linearly, quadratically, and cubically decreased the EE values (*p* < 0.001) but did not influence the moisture, CP, or ash contents of both breast and thigh meat when compared with the control ones. Dietary RBO supplementation linearly and quadratically, and cubically decreased the triglycerides and cholesterol contents of both breast and thigh meat (*p* < 0.01) compared to the control group. The concentration of meat MDA was significantly lower (*p* < 0.001) for broiler chickens consumed the RBO-diets than the control ones; this was strongly indicated by linear response (*p* < 0.001). On the other hand, the meat (breast and thigh) of broiler chickens consumed the RBO diets had a significantly greater GPx content than those consumed the control diet (*p* < 0.001; linear, *p* < 0.001; quadratic, *p* < 0.05, cubic, *p* < 0.05).

Fatty acid composition of the breast and thigh muscles is shown in Table 8. The inclusion of RBO to broiler diets resulted in a decrease (*p* < 0.01, linear, *p* < 0.01) in SFA content and an increase in the MUFA, PUFA, n-6 FA and n-3 FA, in breast meat of the RBO_1.5%_ and RBO_2%_ groups, compared to the control group. Furthermore, a significant elevation (*p* < 0.001) was detected for the n-6:n-3 FA (linear and quadratic, *p* < 0.001), MUFA:SFA (linear and quadratic, *p* < 0.001), and PUFA:SFA (linear, *p* < 0.001) in the groups that contained RBO compared to control. Concerning the thigh meat, there was also a significant decrease (*p* < 0.001) in SFA content, while there was an enhancement (*p* < 0.001) for the MUFA, PUFA, and n-6 FA in the groups supplemented with RBO, this was supported by linear and quadratic responses (*p* < 0.01) compared to the control group. The ratios of n-6 to n-3 FA, MUFA to SFA, and PUFA to SFA were significantly greater (*p* < 0.001, linear, *p* < 0.001) in the thigh meat of the RBO groups than control.

### 3.5. Abdominal Fat Color Parameters

Abdominal fat coloration is presented in Table 9. Color parameters of abdominal fat were not influenced by the feeding of RBO for 35 days.

### 3.6. Liver Lipid Content and MDA

Liver cholesterol, triglycerides, and MDA contents of broiler chickens fed the experimental diets are presented in Table 10. Broiler chickens consumed the RBO diets had significantly lower (*p* < 0.001) liver cholesterol, triglycerides, and MDA contents when compared with those consumed the control diet, this was supported by linear (*p* < 0.001) and quadratic (*p* < 0.05) effects.

### 3.7. Liver Histomorphology

#### 3.7.1. Light Microscope 

**Figure 1:**
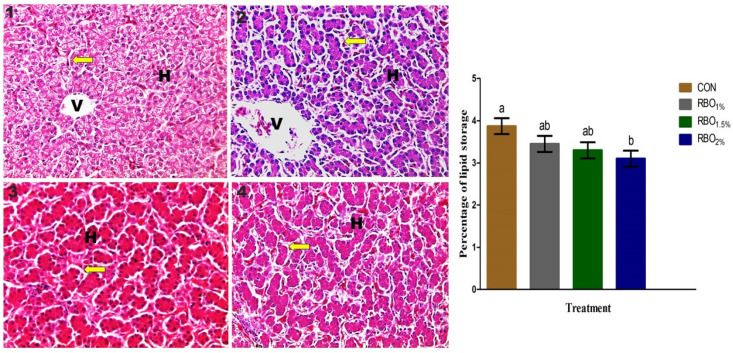
Histological structure of the liver at day 35 of age of broiler chickens fed the basal control diet without RBO (CON, 1), basal diet contained 1% RBO (RBO_1%_, 2), basal diet contained 1.5% RBO (RBO_1.5%_, 3), or basal diet contained 2% RBO (RBO_2%_, 4). Histomicrograph of the liver showing normal liver structure among the treatment groups. H, hepatocytes; V, central vein. Yellow arrows represent hepatic sinusoids between hepatocytes (H & E X100). Percentage of lipid storage estimated via histological method, ^a,b^ means with different letters are varied at *p* < 0.05. Data are shown as mean ± SEM.

Figure 1 presents the photomicrographs of H & E stained liver sections and the histological measurements of broiler chickens fed the control and RBO-supplemented groups. Overall, the histological structures of the liver in all the treatment groups were normal. No pathological lesions or inflammatory changes were detected in the liver of the studied broiler chickens. The liver surface is covered by a layer of thin connective tissue. The hepatocytes were arranged in clusters. The cytoplasm was acidophilic without cytoplasmic vacuoles. Lymphocytes’ aggregations were typically gathered around the portal area, the central veins, and among the hepatic plates. The parenchyma mainly composed of rows of conically shaped hepatocytes bordering sinusoids. The hepatocytes were appeared in a hexagonal design creating hepatic plates. The plates were looked irregularly from the edge of each hepatic lobe to the central vein. The hepatic sinusoids were lined by flat endothelial cells and large von kupffer cells. The percentage of hepatic lipid storage were linearly and quadratically decreased (*p* < 0.01) in the RBO-supplemented groups compared to the control ones. 

#### 3.7.2. Electron Microscope 

**Figure 2:**
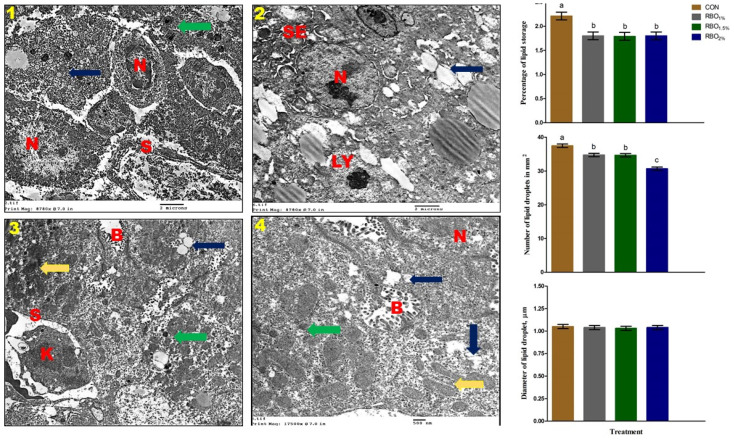
Transmission electron microscope of the liver at day 35 of broiler chickens fed the basal control diet without RBO (CON, 1), basal diet contained 1% RBO (RBO_1%_, 2), basal diet contained 1.5% RBO (RBO_1.5%_, 3), or basal diet contained 2% RBO (RBO_2%_, 4). B, bile canaliculi; S, sinusoids; K, Kupffer cell; N, nucleus of hepatocytes; SE, smooth endoplasmic reticulum; and Ly, lysosomes. Green, yellow, and blue arrows represent glycogen granules, shows mitochondria, and lipid droplet, respectively. Percentage of lipid storage, number and diameter of lipid droplets estimated via histological method, ^a–c^ means with different letters are varied at *p* < 0.05. Data are shown as mean ± SEM.

Figure 2 shows the ultra-structure and the electron microscope measurements of the liver at day 35 of age of the broiler chickens. The ultra- structure of the liver of the ross chickens, at 35 days of age, showed that the hepatic parenchyma was arranged into anastomosing plates of two cell layers or in acinus like arrangement of four or more cells in cross sections. The free surface of the hepatocytes faced the sinusoids, while the central region of the plates contained the bile canaliculi formed by the involvement of two or more hepatocytes. The hepatocyte is appeared as a polygonal shape, with a round nucleus containing one or more nucleoli. The cytoplasm contains some cytoplasmic organelles as mitochondria are numerous, oval to elongate in shape, surrounded by flattened cisterna of rough endoplasmic reticulum. The smooth endoplasmic reticulum appeared as isolated circular area lying scattered throughout the cellular cytoplasm. Few glycogen granules appeared and some lysosome and lipid droplets were observed in the cellular cytoplasm. The diameters of the hepatic lipid droplets were similar in all the treatment groups. However, the percentage of hepatic lipid and the number of lipid droplets were linearly and quadratically reduced (*p* < 0.001) in the liver of broiler chickens fed the RBO diets compared to those fed the control diet.

## 4. Discussion

The demands for healthy foods, such as nutrient-enriched animal products with PUFA and natural antioxidants, are rising. In addition, there is an increasing awareness among consumers about accessing the quality of animal products. RBO is considered one of the healthiest vegetable oils owing to its ideal FA composition and the presence of bioactive components such as γ-oryzanols, tocopherols, tocotrienols, polyphenols, sterols, and phenolic acids [11,12]. These bioactive components were reported to decrease the oxidative stress, blood cholesterol, and LDL levels in hyperlipidemic human subjects [31] or animal models [32,33], and they suggested that RBO may decrease the risk factors of cardiovascular diseases. Therefore, RBO can be an appropriate candidate as a natural source of PUFA and antioxidant constituents in the broilers’ diets to enhance the production performance, health status, antioxidant capacity, and product quality. 

In our study, there was an improvement of the final LW and total WG of the RBO groups compared to the control group. Our finding revealed that between the three RBO groups, a greater inclusion level (RBO_2%_) resulted in higher total WG and greater average daily WG as well as better FCR. However, daily FI of broilers during the entire experimental period was decreased by the inclusion of RBO in diets. The beneficial impacts of RBO on final LW, WG, and FCR were reported previously by Anitha et al. [34], which was enhanced when it included up to 3% in the diets of broilers. Similar findings concerning the FCR and LW enhancement of broilers were achieved by using 2% RBO [17]. However, these earlier studies reported no significant difference in FI among the experimental groups [17,34]. The beneficial effects of RBO on the broiler’s performance may be due to its bioactive components, including γ-oryzanols, tocopherols, tocotrienols, polyphenols, sterols, and ferulic acid [12,17]. In previous studies, vegetable oil reduced the passage rate of feed in the gut, which permits more time for superior nutrient absorption and utilization [35,36], leading to a more effective utilization of nutrients from diet. The reduction in FI noticed herein of broiler chickens fed RBO may be attributed to PUFA and its high energy-yielding capacity [8,35,37]. Attia et al. [8] observed that the inclusion of vegetable oils rich in SFA, i.e., palmitic and stearic acids in the diets of broilers increased their FI when compared with the PUFA-enrich oils, i.e., linolenic and linoleic acids. Similarly, it was reported that SFA has low digestibility compared to UFA, especially during the initial stage of life [8,35,37]. 

The carcass parameters, determined at day 35 of age, showed that broiler chickens who were fed the RBO-enriched diets had a greater dressing percentage and breast yield, but a lower abdominal fat per cent compared to the control ones. These findings suggested that RBO augments the availability of energy for muscle development, whereas the reduce in abdominal fat per cent in the RBO groups indicates a shifting in energy expenditure for meat growth instead of an accumulation in the body, in particular the abdominal cavity [8]. It has been demonstrated that the sources and levels of PUFA considerably affected the carcass traits of broilers [8,35]. The dietary inclusion of oils rich in PUFA was reported to improve the oxidation and decrease FA synthesis, with a resultant reduction of abdominal fat accumulation in broiler chickens [38,39]. At a molecular level, Ahmed et al. [40] reported that RBO treatment overwhelmed the elevated hepatic de novo lipogenesis of the insulin resistance rats via downregulation of lipogenic genes, i.e., superoxide dismutase and catalase. Accordingly, the suppressing effect of RBO may be owing to its un-saponifiable components, which was also found to downregulate sterol regulatory element binding protein-1 [41]. All together, these results suggest that RBO may have a role in the lipid metabolism of broiler chickens; however, further investigations are required to confirm this mechanism. 

Diet strategy is considered one of the documented approaches to augment the immune response in broiler chickens. The thymus gland, spleen, and bursa of Fabricius are the main lymphoid organs in poultry. The measurements of the lymphoid organs’ weights and immune response have been usually applied to evaluate the general immune status of birds [42,43]. In the present study, RBO linearly increased the relative weights of the immune organs, improved serum total protein, and globulin, enhanced HIND antibody titer, and augmented phagocytic activity of the RBO-broilers, within the normal reported values, compared to control. It has been documented that the relative weights of lymphoid organs reveal the growth and function of the immune system, the humoral and cellular immunity [42,43]. Accordingly, an increase in the weights of these organs can imply an immunocompetence [42,43,44]. PUFA induces an immunomodulatory impact via influencing the intercellular signals which alter the leukocytes response due to antigenic stimulation [44]. The FA composition rather than the particular dietary lipids is necessary to augment the cellular and humoral immunity in broilers [8,45]. Linoleic acid, n-6 FA, was reported to be associated with an increase in the lymphocyte proliferation, immune organs size, and antibody titer in mice [46] and in broiler chickens [8,17]. RBO contain a considerable amount of linoleic acid (approximately 36%) and could be responsible for the observed immunomodulatory effect in the RBO groups. On the other hand, some plant components can augment the multiplication of advantageous microbiota, while diminishing the proliferation of harmful ones, consequently indirectly boosting host’s immunity [47]. The flavonoids and phenolic compounds are known for their antioxidant, anti-inflammatory, and antimicrobial activities [48]. Since RBO is rich in flavonoids and phenolic compounds, in the present study, possibly dietary supplementation with RBO notably enhanced the immune status of broiler chickens.

Analysis of serum enzyme activities is an imperious procedure in the assessment of poultry health problems. Enzymes, such as ALT and AST, are generally existing with high concentrations in the liver [49] and are considered as particular indicators of hepatic cellular damage and inflammation [50]. ALP is an enzyme produced in all tissue types and is mainly responsible for dephosphorylation of a substrate and activated in alkaline pH. Its raised blood values are commonly noted in hepatic damages [49,50]. In our study, RBO supplementation linearly reduced the serum AST, ALT, and ALP when compared with the control group. The current findings indicate that RBO supplementation did not induce any detrimental effects on the liver. Lower serum AST, ALT, and ALP levels, within the normal values, may probably indicate that RBO decreased tissue damage and enhanced liver functions in the RBO broilers, since higher activities of these markers were reported to be associated with liver damage and inflammation [51]. Along with serum biochemical constituents, histological structure can be used to determine liver abnormalities and help in assessing disease diagnosis in poultry. In support of our findings, the general histological structure and ultrastructural of the liver of the experimental groups are normal and comparable to previous observations of the birds’ liver [43,52]. No pathological lesions were observed in the broiler chickens’ livers of the current experiment. The lipid content of the experimental groups whether assessed using images from light or electron microscope revealed that RBO feeding linearly decreased the percentage of hepatic lipid storage and number of lipid droplets compared to control. Regarding the hepatic chemical assessments, the broiler chickens consumed the RBO diets had significantly lower liver cholesterol, triglycerides, and MDA contents when compared with those consumed the control diet. The liver plays a central role in lipogenesis in birds, and contrary to mammals, the fat synthesis occurs mainly in the liver and is restricted in the adipose tissue [53]. Based on the obtained findings, we suggested that RBO can improve liver lipid profile and induce liver protective effects through its phenolic and antioxidant compounds [32,33]. The presence of phenolic compounds and antioxidants allows RBO to protect hepatic tissue from lipid peroxidation and thus decreased serum levels of ALT, AST, and ALP. Previous studies by Wang et al. [32] and Zhang et al. [33] showed that rice bran phenolic extract exerts its hypolipidemic effect through activation of AMP-activated protein kinase, wherein phenolic compounds found in rice bran such as p-coumaric acid, ferulic acid, and rutin play a fundamental role. 

It is worth noting that RBO supplementation resulted in a reduction in serum levels of total lipids, total cholesterol, LDL, and triglycerides, while it increased serum HDL level. The favorable actions of RBO on total lipids, total cholesterol, and LDL are possibly due to its richness in non-saponifiable components, including sterols (b-sitosterol, campesterol and stigmasterol), c-oryzanol, and tocotrienols. Lichtenstien et al. [54] showed a higher content of b-sitosterol, campesterol and stigmasterol in RBO than other vegetable oils. Plant sterols are observed to be accountable for a 30–40% reduction in the cholesterol absorption [55]. Furthermore, oryzanol and tocotrienols found in RBO lessen the rate of endogenous cholesterol synthesis through diminishing the HMG-CoA reductase enzyme [32,56], and increasing the expression of cholesterol 7-alpha-hydroxylase, which is the rate-limiting enzyme in the synthesis of bile acids from cholesterol. Another suggested mechanism accountable for the reduction of total cholesterol due to feeding RBO is its omega-3 FA content which can inhibit the apolipoprotein-B100 and LDL synthesis and accordingly result in a decrease in total cholesterol [54,57]; however, the exact mechanism in broiler chickens is still unclear and necessitates further investigations. 

Meat color is one of the critical indicators of freshness and wholesomeness of any meat product, relying on it, customers accomplish an initial impression of the product [58]. Numerous factors were reported to influence the color of broiler’s meat including genetics, sex, age, diet composition, the heme pigments, and pre-slaughter condition [58]. In the current study, dietary RBO had no influence on L* (lightness), a* (redness), and b* (yellowness) values of breast or thigh meat at 24 h post-mortem among the experimental groups. The present findings are in line with the reports of Khatun et al. [59] and Jankowski et al. [60], who found that meat color was not influenced by the dietary oils supplementation. However, these results contradict the observations of Turcu et al. [5] and Qi et al. [61] and who found that dietary supplementation with various oils influenced the meat color of broiler chickens. The inconsistency between these studies may be due to various types of the studied plant oils. Measurement of the meat pH is vital because there is a relationship between pH and physicochemical properties of the meat, such as color and hardness [5]. In this study, the pH values of the breast and thigh muscles are within the ranges of normal pH limits [5]. Our results are in agreement with those of Khatun et al. [59] and Jankowski et al. [60] who observed no differences in the meat pH at 24 h post-mortem in broiler chickens fed various dietary oils. In goose, the inclusion of full-fat rice bran at 6–18% did not influence the meat quality traits, involving color parameters, and pH [62]. The lack of differences observed in meat color in the current trial might be owing to the similarity in the pH values for all groups. Furthermore, in this trial, the lack of variances between the experimental groups for the pH of the meat could be owing to the fact that RBO had no effects on the glycogen content of the meat. Glycogen plays a key role in the pH value of meat [63]; nevertheless, the meat glycogen content was not measured in the present trial, which necessitates further research. On the other hand, there was non-significant difference in broiler abdominal fat coloration in the present study, which is probably due to the low contents of beta-carotene, lutein, and zeaxanthin found in RBO [64]. To our knowledge, there have been no previous reports on the effects of dietary RBO on broiler meat or abdominal fat colorations. 

It is well acknowledged that lipid peroxidation is one of the major processes that affect the quality of meat and its products [65]. The inclusion of RBO at different levels in the broiler diets has been performed to decrease the lipid content while increase the antioxidant contents of the meat in order to avoid its quality deterioration. In the present trial, the antioxidant effect of RBO on meat lipid peroxidation was noticed both in thigh and breast meat, compared to control samples. Dietary inclusion of RBO linearly and quadratically improved the meat GPx content, while reduced its EE %, triglycerides, cholesterol, and MDA contents. On the other hand, with the increasing RBO level there was no variation in CP content in the broiler meat, which may indicate that RBO had no influence on protein synthesis in the meat. In broiler chickens, in ovo injection of RBO increased the GPx content but decreased the MDA content in the breast and heart muscles [66]. Full fat rice bran inclusion linearly and quadratically reduced crude fat content in goose meat but did not affect the meat CP content [62]. The γ-oryzanol, tocopherols, and tocotrienols components of RBO are thought as the major ingredients responsible for the hypolipidemic and antioxidant activities of RBO [67]. Although the mechanism underlying this effect is not clear at the moment, oryzanol, and tocopherols of RBO are supposed to be the main cause for this beneficial outcome. Furthermore, phenolic compounds (e.g., polyphenols) induced the antioxidant potential which may diminish the formation of free radicals and subsequently counteract the peroxidation of PUFA [68]. 

Chicken meat is considered as good sources of protein and essential FA for humans. Diet manipulation has a considerable influence on the broiler meat composition. Meat FA composition is considered as a precious indicator of meat quality from the human health standpoint. Vegetable oils are valuable dietary constituents as a source of FA that can possibly be redirected in the poultry products [65]. Interestingly, our findings revealed that the inclusion of RBO in the broiler diets increased the meat MUFA, PUFA, n-6, and n-3 FA contents, but decreased the SFA level. The improvement of PUFA in the meat of RBO groups chiefly caused by the diminution of SFA and the elevation of C18:2n-6 and C18:3n-3 FA in the meat. The most effective of RBO supplementation was the enrichment of the breast meat with PUFA, n-6 FA, n-3 FA, and PUFA:SFA, and lowering the SFA content. PUFA show an important role in lessening the occurrence of cardiovascular and inflammatory diseases in humans [69]. To our knowledge, there is inadequate data in the literature regarding the dietary inclusion of RBO and the modification of the meat FA composition in poultry. According to Turcu, et al. [5] and Attia et al. [8], the dietary supplementation with PUFA-rich oils in broiler diets resulted in a modification in the meat FA profile, namely enrichment the meat with PUFA and a decrease in SFA level. Sun et al. [62] showed that full-fat rice bran inclusion decreased MUFA and increased PUFA level in the goose meat. In pigs, Jayaraman et al. [70] reported that the inclusion full-fat rice bran as a replacement of corn in their diets linearly decreased SFA (palmitic and stearic acids), but increased n-6 FA and n-3 FA in meat. The hypolipidemic action of the phenolic acids presented in RBO may be contributed to the enhancement of the FA profile of broiler meat through lipid homeostasis. Previous researchers have proposed that the FA content of poultry meat could be modified through dietary manipulation by adding plant oils and antioxidants [5,8,17,62,68], which in turn could extend the shelf-life and improve the quality of meat products. 

## 5. Conclusions

Under the condition of the current experiment, RBO inclusion as a source of PUFA and natural antioxidants had a positive effect on the growing performance, dressing percentage, and immune status. Furthermore, RBO supplementation decreased the abdominal fat yield and EE content in the meat and increased the meat PUFA without influencing other meat quality traits such as color and pH, which may be beneficial for consumers. RBO improved the antioxidant capacity and lipid profile of the meat and the liver. In conclusion, RBO could be used as a valuable efficient ingredient in broiler chickens’ diets to stimulate the growth performance and immune status, enhance the antioxidant activity and blood lipid profile, augment liver function, and improve the nutritive value of the meat.

## Figures and Tables

**Table 1:** Ingredients and chemical composition of the experimental diets ^1^.

Items, g/kg	Starter (d 1 to 10)				Grower (d 11 to 24)				Finisher (d 25 to 35)			
	CON	RBO_1%_	RBO_1.5%_	RBO_2%_	CON	RBO_1%_	RBO_1.5%_	RBO_2%_	CON	RBO_1%_	RBO_1.5%_	RBO_2%_
Yellow corn	560.0	560.0	560.0	560.0	602.6	602.6	602.6	602.6	647.9	647.9	647.9	647.9
SBM, 46% CP	319.0	319.0	319.0	319.0	284.0	284.0	284.0	284.0	230.0	230.0	230.0	230.0
Corn gluten, 62% CP	60.0	60.0	60.0	60.0	55.0	55.0	55.0	55.0	65.0	65.0	65.0	65.0
Soybean oil	20.0	15.0	10.0	0	20.0	15.0	10.0	0.0	20.0	15.0	10.0	0.0
RBO	0.0	5.0	10.0	20.0	0	5.0	10.0	20.0	0.0	5.0	10.0	20.0
Limestone	12.0	12.0	12.0	12.0	16.3	16.3	16.3	16.3	15.2	15.2	15.2	15.2
Dicalcium phosphate	17.3	17.3	17.3	17.3	11.1	11.1	11.1	11.1	10.5	10.5	10.5	10.5
L-Lysine ^a^	2.34	2.34	2.34	2.34	1.86	1.86	1.86	1.86	2.41	2.41	2.41	2.41
DL-Methionine ^b^	1.49	1.49	1.49	1.49	1.24	1.24	1.24	1.24	1.11	1.11	1.11	1.11
Common salt	2.5	2.5	2.5	2.5	2.5	2.5	2.5	2.5	2.5	2.5	2.5	2.5
Premix ^c^	3.0	3.0	3.0	3.0	3.0	3.0	3.0	3.0	3.0	3.0	3.0	3.0
Na-bicarbonate	2.4	2.4	2.4	2.4	2.4	2.4	2.4	2.4	2.4	2.4	2.4	2.4
Calculated chemical composition												
ME, MJ/kg	12.55	12.55	12.55	12.55	12.95	12.95	12.95	12.95	13.30	13.30	13.30	13.30
CP	231.5	231.5	231.5	231.5	215.9	215.9	215.9	215.9	201.1	201.1	201.1	201.1
Calcium	9.6	9.6	9.6	9.6	8.7	8.7	8.7	8.7	8.1	8.1	8.1	8.1
Available P	4.8	4.8	4.8	4.8	4.4	4.4	4.4	4.4	4.1	4.1	4.1	4.1
Lysine	14.4	14.4	14.4	14.4	12.9	12.9	12.9	12.9	11.9	11.9	11.9	11.9
Methionine	5.6	5.6	5.6	5.6	5.1	5.1	5.1	5.1	4.8	4.8	4.8	4.8
Analyzed chemical composition												
DM	911.7	911.6	911.8	911.5	909.7	908.9	900.6	909.5	912.5	913.4	912.7	912.4
CP	230.6	230.4	230.5	230.8	214.9	215.3	215.0	215.5	200.0	199.9	200.8	200.6
EE	36.2	36.1	36.0	36.2	35.4	35.3	35.6	35.9	36.1	36.0	36.4	36.2
CF	45.4	45.7	45.8	45.3	44.9	45.1	44.9	45.6	45.2	45.0	45.3	45.7
Ash	59.8	59.8	60.2	60.0	59.8	59.2	59.5	59.8	61.0	59.9	61.2	61.1

^1^ Dietary treatments: CON, basal diet without RBO; RBO_1%_, basal diet contained 1% RBO; RBO_1.5%_, basal diet contained 1.5% RBO; RBO_2%_, basal diet contained 2% RBO. ^a^ Supplied per kg of diet: vit. A, 12,000 IU; vit. D3, 3000 IU; vit. E, 40 mg; vit. K3, 3 mg; vit. B1, 2 mg; vit. B2, 6 mg; vit. B5, 12 mg; vi. B6, 5 mg; vitamin B12, 0.02 mg; vit. B3, 45 mg; vit. B7, 0.075 mg; vit. B9, 2 mg; Mn, 100 mg; Zn, 60 mg; Fe, 30 mg; Cu, 10 mg; I, 1 mg; Se, 0.2 mg; Co, 0.1 mg. ^b^ L-Lysine, lysine monohydrochloride (Feed Grade, 99%). ^c^ DL-Methionine, Met AMINO (DL-2-amino-4-(methyl-thio)-butane acid, DL-Met, α-amino-Y-methyl-oily acid) (Feed Grade, 99%).

**Table 2:** Fatty acid composition (weight %) and γ-oryzanol of rice bran oil (RBO) and soybean oil (SBO).

Items ^1^	RBO	SBO	
C14:0	Myristic	0.47	ND
C16:0	Palmitic	17.64	10.59
C17:0	Heptadecanoic	0.30	0.12
C18:0	Stearic	0.96	4.01
C20:0	Arachidic	0.67	0.40
C16:1	Palmitoleic	0.41	0.21
C17:1	Heptadecenoic	0.18	0.09
C18:1	Oleic	40.81	28.66
C20:1	Eicosenoic	0.71	0.3
C18:2	Linoleic	36.15	51.27
C18:3	Linolenic	1.85	4.98
SFA		20.04	15.12
MUFA		41.96	28.63
PUFA		38.00	56.25
γ-oryzanol (g/100 g oil)		3.58	-

^1^ SFA, saturated fatty acids; MUFA, monounsaturated fatty acids; PUFA, polyunsaturated fatty acids. Data were collected from Agricultural Research Center, Egypt.

**Table 3:** Growth performance of broiler chickens fed diets contained graded level of rice bran oil (RBO) during the experiment (1–35 days of age).

Items ^2^	Treatments ^1^				SEM	*p*-Value	Contrasts		
	CON	RBO_1%_	RBO_1.5%_	RBO_2%_			Linear	Quadratic	Cubic
Initial BW (0 d), g	43.00	42.95	43.05	42.95	0.264	0.978	0.95	0.89	0.676
1–21 day of age									
LW (21 d), g	735.79 ^c^	775.06 ^c^	853.67 ^b^	948.04 ^a^	17.932	<0.001	<0.001	0.03	0.679
Total WG, g	692.79 ^c^	732.11 ^c^	810.62 ^b^	905.09 ^a^	17.89	<0.001	<0.001	0.03	0.63
Daily WG, g	32.99 ^c^	34.86 ^c^	38.60 ^b^	43.10 ^a^	1.85	<0.001	<0.001	0.03	0.68
Daily FI, g	53.56 ^a^	50.29 ^b^	49.60 ^b^	50.98 ^b^	1.83	<0.001	<0.001	<0.001	<0.001
FCR	1.63 ^a^	1.45 ^b^	1.29 ^c^	1.19 ^c^	0.041	<0.001	<0.001	0.19	0.71
22–35 day of age									
LW (35 d), g	2065.2 ^c^	2137.5 ^b^	2236.2 ^a^	2264.5 ^a^	16.47	<0.001	<0.001	0.06	0.06
Total WG, g	1329.4	1362.4	1382.5	1347.7	27.16	0.06	0.83	0.01	0.39
Daily WG, g	94.96	97.32	98.75	94.03	1.94	0.06	0.83	0.01	0.39
Daily FI, g	163.80 ^a^	159.32 ^a^	159.53 ^a^	144.91 ^b^	3.50	<0.001	<0.001	<0.001	<0.001
FCR	1.73 ^a^	1.65 ^ab^	1.62 ^bc^	1.54 ^c^	0.034	<0.001	<0.001	0.84	0.31
1–35 day of age									
Total WG, g	2022.2 ^c^	2094.5 ^b^	2193.1 ^a^	2221.6 ^a^	16.42	<0.001	<0.001	0.06	0.06
Daily WG, g	57.77 ^b^	59.84 ^b^	62.66 ^ab^	63.47 ^a^	1.50	<0.001	<0.001	0.06	0.07
Daily FI, g	106.23 ^a^	99.62 ^b^	93.86 ^c^	88.55 ^c^	2.53	<0.001	<0.001	0.07	0.80
FCR	1.69 ^a^	1.57 ^b^	1.49 ^c^	1.39 ^d^	0.016	<0.001	<0.001	0.31	0.18

^a–d^ Means within the same row having different letters are varied at *p* < 0.05; Tukey’s tests were applied to compare means; SEM = Standard error of the mean. ^1^ Dietary treatments: CON, basal diet without RBO; RBO_1%_, basal diet contained 1% RBO; RBO_1.5%_, basal diet contained 1.5% RBO; RBO_2%_, basal diet contained 2% RBO. ^2^ LW, live weight; WG, weight gain; FI, feed intake; FCR, feed conversion ratio.

**Table 4:** Carcass traits (% of live body weight) and organs yields of broiler chickens fed diets contained graded level of rice bran oil (RBO) at 35 days of age.

Items	Treatments ^1^				SEM	*p*-Value	Contrasts		
	CON	RBO_1%_	RBO_1.5%_	RBO_2%_			Linear	Quadratic	Cubic
Slaughter parameter									
Dressing, %	73.34 ^c^	75.65 ^b^	76.07 ^b^	77.98 ^a^	0.460	<0.001	<0.001	0.039	0.006
Muscle yields									
Breast yield, %	29.88 ^c^	30.27 ^bc^	31.49 ^ab^	31.85 ^a^	0.427	<0.001	0.01	0.007	<0.001
Thigh yield, %	21.70	21.85	21.37	21.91	0.509	0.743	0.96	0.33	0.65
Organ yields									
Liver, %	2.51	2.56	2.55	2.53	0.140	0.50	0.23	0.12	0.38
Heart, %	0.47	0.50	0.47	0.49	0.031	0.11	0.73	0.42	0.30
Gizzard, %	2.55	2.56	2.58	2.59	0.210	0.62	0.52	0.18	0.29
Abdominal fat, %	2.38 ^a^	1.75 ^b^	1.72 ^b^	1.70 ^b^	0.190	0.014	0.003	0.002	0.72
Immune organs									
Spleen, %	0.070 ^b^	0.071 ^b^	0.091 ^a^	0.089 ^a^	0.002	<0.001	<0.001	0.23	0.54
Thymus gland, %	0.097 ^d^	0.102 ^c^	0.112 ^b^	0.126 ^a^	0.003	<0.001	<0.001	0.06	0.63
Bursa of Fabricius, %	0.094 ^d^	0.111 ^c^	0.122 ^b^	0.137 ^a^	0.002	<0.001	<0.001	0.44	0.17

^a–d^ Means within the same row having different letters are varied at *p* < 0.05; Tukey’s tests were applied to compare means; SEM = Standard error of the mean. ^1^ Dietary treatments: CON, basal diet without RBO; RBO_1%_, basal diet contained 1% RBO; RBO_1.5%_, basal diet contained 1.5% RBO; RBO_2%_, basal diet contained 2% RBO.

**Table 5:** Blood biochemical constituents of broiler chickens fed the experimental diets at 35 days of age.

Items ^2^	Treatments ^1^				SEM	*p*-Value	Contrasts		
	CON	RBO_1%_	RBO_1.5%_	RBO_2%_			Linear	Quadratic	Cubic
Protein metabolites									
Total protein, g/dL	4.18 ^b^	4.50 ^b^	5.06 ^a^	4.96 ^a^	0.137	<0.001	<0.001	0.04	0.05
Albumin, g/dL	3.06	3.16	3.40	3.46	0.148	0.06	0.11	0.85	0.50
Globulin, g/dL	1.12 ^b^	1.34 ^ab^	1.66 ^a^	1.50 ^a^	0.122	0.003	0.002	0.04	0.15
A/G	2.79	2.41	2.08	2.35	0.272	0.12	0.07	0.12	0.55
Lipid metabolites									
Total lipids, mg/dL	664.95 ^a^	518.30 ^b^	424.67 ^c^	405.68 ^d^	4.794	<0.001	<0.001	<0.001	0.11
Total cholesterol, mg/dL	150.39 ^a^	136.52 ^b^	128.37 ^c^	119.94 ^d^	2.444	<0.001	<0.001	0.03	0.23
Triglycerides,	126.94 ^a^	118.55 ^b^	97.77 ^d^	103.33 ^c^	1.784	<0.001	<0.001	<0.001	<0.001
LDL, mg/dL	52.47 ^a^	39.70 ^b^	21.74 ^d^	33.56 ^c^	0.552	0.02	0.01	0.05	0.18
HDL, mg/dL	82.80 ^c^	86.47 ^b^	88.10 ^b^	93.47 ^a^	1.182	<0.001	<0.001	<0.001	<0.001
Liver functions									
AST, U/dL	77.00 ^a^	75.00 ^ab^	71.80 ^b^	73.60 ^b^	1.183	0.003	0.002	0.04	0.12
ALT, U/dL	69.00	67.80	65.80	66.60	1.149	0.06	0.02	0.24	0.34
ALP, U/L	12.34	11.22	11.14	11.52	0.439	0.06	0.09	0.03	0.68
Kidney functions									
Creatinine, mg/dL	0.52	0.53	0.47	0.50	0.190	0.20	0.51	0.46	0.22
Uric acid, mg/dL	5.56	5.52	5.60	5.70	0.332	0.12	0.34	0.34	0.31
Antibody titer									
HIND, log^2^	3.80 ^c^	4.80 ^b^	5.40 ^b^	6.00 ^a^	0.201	<0.001	<0.001	0.59	0.34
HIAI, log^2^	4.00	3.80	4.60	4.40	0.351	0.15	0.09	0.24	0.09
HIBD, log^2^	3.40	3.20	3.80	3.60	0.316	0.29	0.24	0.30	0.12
Phagocytic activity									
Phagocytic index	3.57 ^b^	3.68 ^b^	4.18 ^ab^	4.84 ^a^	0.390	0.02	0.03	0.18	0.13
Phagocytic activity	61.80 ^b^	65.80 ^ab^	66.80 ^ab^	71.40 ^a^	2.577	0.01	0.02	0.13	0.17

^a–d^ Means within the same row having different letters are varied at *p* < 0.05; Tukey’s tests were applied to compare means; SEM = Standard error of the mean. ^1^ Dietary treatments: CON, basal diet without RBO; RBO_1%_, basal diet contained 1% RBO; RBO_1.5%_, basal diet contained 1.5% RBO; RBO_2%_, basal diet contained 2% RBO. ^2^ A/G, albumin to globulin ratio; LDL, low density lipoprotein; HDL, high density lipoprotein; AST, aspartate aminotransferase; ALT, alanine aminotransferase; ALP, alkaline phosphatase; HIND, Hemagglutination-inhibition test for Newcastle disease; HIAI, Hemagglutination-inhibition test for Avian Influenza; HIBD, Hemagglutination-inhibition test for bursa disease.

**Table 6:** Breast meat physical, chemical, lipid and antioxidant characteristics of broiler chickens fed the experimental diets at 35 days of age.

Items ^2^	Treatments ^1^				SEM	*p*-Value	Contrasts		
	CON	RBO_1%_	RBO_1.5%_	RBO_2%_			Linear	Quadratic	Cubic
Physical characteristics									
pH	6.10	6.07	6.07	6.11	0.019	0.11	0.56	0.20	0.97
Color									
L*	55.96	52.86	55.73	55.48	1.502	0.10	0.80	0.20	0.03
a*	3.02	3.27	3.16	3.40	0.497	0.22	0.28	0.12	0.56
b*	11.28	12.05	11.89	11.76	0.708	0.60	0.56	0.30	0.56
Proximate chemical composition									
Moisture, %	71.06	71.04	71.54	71.67	0.752	0.006	0.11	0.98	0.24
CP, %	24.40	24.50	24.30	24.21	0.591	0.12	0.17	0.13	0.27
EE, %	2.72 ^a^	2.74 ^a^	2.20 ^b^	2.29 ^b^	0.064	<0.001	<0.001	<0.001	<0.001
Ash, %	1.82	1.70	1.94	1.83	0.159	0.43	0.64	0.70	0.35
Lipid content									
Triglycerides, mg/100 g	57.84 ^a^	44.15 ^b^	40.44 ^c^	40.39 ^c^	0.581	<0.001	<0.001	<0.001	0.009
Cholesterol, mg/100 g	88.87 ^a^	70.20 ^b^	44.32 ^c^	47.10 ^c^	0.563	<0.001	<0.001	<0.001	<0.001
Antioxidant capacity									
GPx, U/g	0.77 ^d^	1.13 ^c^	1.40 ^b^	2.02 ^a^	0.057	<0.001	<0.001	0.01	0.04
MDA, mg/kg	0.86 ^a^	0.71 ^b^	0.62 ^c^	0.38 ^d^	0.013	<0.001	<0.001	0.001	0.003

^a–d^ Means within the same row having different letters are varied at *p* < 0.05; Tukey’s tests were applied to compare means; SEM = Standard error of the mean. ^1^ Dietary treatments: CON, basal diet without RBO; RBO_1%_, basal diet contained 1% RBO; RBO_1.5%_, basal diet contained 1.5% RBO; RBO_2%_, basal diet contained 2% RBO. ^2^ L*, lightness; a*, redness; b*, yellowness; CP, crude protein; EE, ether extract; GPx, glutathione peroxidase; MDA, malondialdehyde.

**Table 7:** Thigh meat physical, chemical, lipid and antioxidant characteristics of broiler chickens fed the experimental diets at 35 days of age.

Items ^2^	Treatments ^1^				SEM	*p*-Value	Contrasts		
	CON	RBO_1%_	RBO_1.5%_	RBO_2%_			Linear	Quadratic	Cubic
Physical characteristics									
pH	6.25	6.22	6.15	6.22	0.023	0.25	0.42	0.34	0.13
Color									
L*	55.59	54.34	54.53	55.77	1.428	0.73	0.89	0.27	0.93
a*	2.76	3.23	3.43	3.25	0.305	0.15	0.18	0.26	0.57
b*	11.73	12.31	11.81	12.34	0.690	0.36	0.95	0.72	0.07
Proximate chemical composition									
Moisture, %	72.04	72.08	72.53	72.50	0.712	0.69	0.20	0.70	0.61
CP, %	21.62	21.86	22.22	22.58	0.802	0.19	0.25	0.36	0.65
EE, %	5.25 ^a^	5.00 ^a^	4.15 ^b^	3.83 ^b^	0.15	<0.001	<0.001	<0.001	<0.001
Ash, %	1.08	1.06	1.09	1.09	0.07	0.32	0.41	0.59	0.19
Lipid content									
Triglycerides, mg/100 g	69.22 ^a^	59.55 ^b^	60.92 ^b^	51.95 ^c^	0.681	<0.001	<0.001	<0.001	<0.001
Cholesterol, mg/100 g	177.27 ^a^	162.71 ^b^	139.68 ^c^	115.03 ^d^	2.205	<0.001	<0.001	0.01	0.35
Antioxidant capacity									
GPx, U/g	0.78 ^d^	0.94 ^c^	1.30 ^b^	1.61 ^a^	0.021	<0.001	<0.001	0.002	0.007
MDA, mg/kg	0.89 ^a^	0.74 ^b^	0.51 ^c^	0.44 ^c^	0.043	<0.001	<0.001	0.25	0.14

^a–d^ Means within the same row having different letters are varied at *p* < 0.05; Tukey’s tests were applied to compare means; SEM = Standard error of the mean. ^1^ Dietary treatments: CON, basal diet without RBO; RBO_1%_, basal diet contained 1% RBO; RBO_1.5%_, basal diet contained 1.5% RBO; RBO_2%_, basal diet contained 2% RBO. ^2^ L*, lightness; a*, redness; b*, yellowness; CP, crude protein; EE, ether extract; GPx, glutathione peroxidase; MDA, malondialdehyde.

**Table 8:** Meat fatty acid profile (% total fatty acids) of broiler chickens fed the experimental diets at 35 days of age.

Items ^2^	Treatments ^1^				SEM	*p*-Value	Contrasts		
	CON	RBO_1%_	RBO_1.5%_	RBO_2%_			Linear	Quadratic	Cubic
Breast meat									
SFA	35.00 ^a^	34.29 ^a^	32.12 ^b^	31.22 ^b^	0.286	<0.001	<0.001	0.65	0.02
MUFA	38.17 ^c^	38.82 ^bc^	39.83 ^a^	39.37 ^ab^	0.269	0.001	0.001	0.02	0.06
PUFA	26.83 ^b^	26.89 ^b^	28.05 ^ab^	29.41 ^a^	0.433	0.001	<0.001	0.07	0.53
n-6 FA	23.50 ^c^	23.52 ^c^	24.60 ^b^	25.96 ^a^	0.279	<0.001	<0.001	0.002	0.29
n-3 FA	3.33 ^b^	3.37 ^ab^	3.45 ^a^	3.45 ^a^	0.041	0.04	0.009	0.51	0.38
n6:n3	7.06 ^c^	6.98 ^d^	7.13 ^b^	7.52 ^a^	0.01	<0.001	<0.001	<0.001	0.003
MUFA:SFA	1.09 ^d^	1.13 ^c^	1.24 ^b^	1.26 ^a^	0.002	<0.001	<0.001	<0.001	<0.001
PUFA:SFA	0.77 ^c^	0.78 ^c^	0.87 ^b^	0.94 ^a^	0.02	<0.001	<0.001	0.11	0.19
Thigh meat									
SFA	36.02 ^a^	33.85 ^b^	32.77 ^c^	33.62 ^bc^	0.286	<0.001	<0.001	0.001	0.38
MUFA	38.96 ^c^	38.99 ^c^	40.93 ^a^	39.62 ^b^	0.436	<0.001	<0.001	<0.001	<0.001
PUFA	25.02 ^b^	27.16 ^a^	26.30 ^a^	26.76 ^a^	0.327	0.001	0.003	0.007	0.003
n-6 FA	21.65 ^b^	23.74 ^a^	22.92 ^a^	23.50 ^a^	0.269	<0.001	0.001	0.004	0.001
n-3 FA	3.37	3.42	3.38	3.26	0.057	0.10	0.07	0.06	0.95
n6:n3	6.42 ^d^	6.94 ^b^	6.78 ^c^	7.21 ^a^	0.036	<0.001	<0.001	0.12	<0.001
MUFA:SFA	1.08 ^c^	1.15 ^b^	1.25 ^a^	1.18 ^b^	0.009	<0.001	<0.001	<0.001	<0.001
PUFA:SFA	0.69 ^b^	0.80 ^a^	0.80 ^a^	0.79 ^a^	0.015	<0.001	<0.001	0.001	0.06

^a–d^ Means within the same row having different letters are varied at *p* < 0.05; Tukey’s tests were applied to compare means; SEM = Standard error of the mean. ^1^ Dietary treatments: CON, basal diet without RBO; RBO_1%_, basal diet contained 1% RBO; RBO_1.5%_, basal diet contained 1.5% RBO; RBO_2%_, basal diet contained 2% RBO. ^2^ SFA, saturated fatty acids; MUFA, monounsaturated fatty acids; PUFA, polyunsaturated fatty acids; FA, fatty acids.

**Table 9:** Abdominal fat color parameters of broiler chickens fed the experimental diet at 35 days of age.

Items ^2^	Treatments ^1^				SEM	*p*-Value	Contrasts		
	CON	RBO_1%_	RBO_1.5%_	RBO_2%_			Linear	Quadratic	Cubic
L*	70.30	69.84	69.80	69.92	0.741	0.08	0.10	0.25	0.35
a*	6.40	6.81	6.71	6.59	0.365	0.20	0.31	0.16	0.89
b*	26.82	27.21	27.09	27.40	0.625	0.13	0.08	0.67	0.17

Tukey’s tests were applied to compare means; SEM = Standard error of the mean. ^1^ Dietary treatments: CON, basal diet without RBO; RBO_1%_, basal diet contained 1% RBO; RBO_1.5%_, basal diet contained 1.5% RBO; RBO_2%_, basal diet contained 2% RBO. ^2^ L*, lightness; a*, redness; b*, yellowness.

**Table 10:** Liver triglycerides, cholesterol and Malondialdehyde (MDA) concentration of broiler chickens fed the experimental diets.

Items	Treatments ^1^				SEM	*p*-Value	Contrasts		
	CON	RBO_1%_	RBO_1.5%_	RBO_2%_			Linear	Quadratic	Cubic
Cholesterol, mg/g	4.87 ^a^	4.08 ^b^	3.22 ^c^	2.15 ^d^	0.077	<0.001	<0.001	0.04	0.62
Triglycerides, mg/g	7.49 ^a^	6.69 ^b^	6.05 ^c^	5.05 ^d^	0.049	<0.001	<0.001	<0.001	0.35
MDA, mg/kg	1.78 ^a^	0.64 ^b^	0.51 ^b^	0.38 ^b^	0.021	<0.001	<0.001	<0.001	0.58

^a–d^ Means within the same row having different letters are varied at *p* < 0.05; Tukey’s tests were applied to compare means; SEM = Standard error of the mean. ^1^ Dietary treatments: CON, basal diet without RBO; RBO_1%_, basal diet contained 1% RBO; RBO_1.5%_, basal diet contained 1.5% RBO; RBO_2%_, basal diet contained 2% RBO.

## Data Availability

The data presented in this study are available on request from the corresponding author.
